# Arabidopsis Kunitz Trypsin Inhibitors in Defense Against Spider Mites

**DOI:** 10.3389/fpls.2018.00986

**Published:** 2018-07-10

**Authors:** Ana Arnaiz, Lucia Talavera-Mateo, Pablo Gonzalez-Melendi, Manuel Martinez, Isabel Diaz, M. E. Santamaria

**Affiliations:** ^1^Centro de Biotecnología y Genómica de Plantas, Universidad Politécnica de Madrid, Instituto Nacional de Investigación y Tecnología Agraria y Alimentaria, Madrid, Spain; ^2^Departamento de Biotecnología-Biología Vegetal, Escuela Técnica Superior de Ingeniería Agronómica, Alimentaria y de Biosistemas, Universidad Politécnica de Madrid, Madrid, Spain

**Keywords:** plant-herbivore interphase, *Tetranychus urticae*, *Arabidopsis thaliana*, serine protease inhibitors, cysteine protease inhibitors, spider mite digestion

## Abstract

*Tetranychus urticae* (two-spotted spider mite) is a striking example of polyphagy among herbivores with an extreme record of pesticide resistance and one of the most significant pests in agriculture. The *T. urticae* genome contains a large number of cysteine- and serine-proteases indicating their importance in the spider mite physiology. This work is focused on the potential role of the Kunitz trypsin inhibitor (KTI) family on plant defense responses against spider mites. The molecular characterization of two of these genes, At*KTI4* and At*KTI5*, combined with feeding bioassays using T-DNA insertion lines for both genes was carried out. Spider mite performance assays showed that independent *KTI* silencing Arabidopsis lines conferred higher susceptibility to *T. urticae* than WT plants. Additionally, transient overexpression of these inhibitors in *Nicotiana benthamiana* demonstrated their ability to inhibit not only serine- but also cysteine-proteases, indicating the bifunctional inhibitory role against both types of enzymes. These inhibitory properties could be involved in the modulation of the proteases that participate in the hydrolysis of dietary proteins in the spider mite gut, as well as in other proteolytic processes.

## Introduction

The fact that higher plants are sessile organisms has favored the acquisition of sophisticated resources to prevent or hamper pest feeding (Walling, 2000; Wu and Baldwin, 2010). Such defenses can be constitutive and/or induced upon attack by herbivore pests. Induced defenses include morphological and metabolic changes with a negative impact on phytophagous arthropod behavior (Walling, 2000; Howe and Jander, 2008; Alba et al., 2011) or the attraction of natural enemies of the herbivore (Sabelis et al., 1999; Dicke and Baldwin, 2010). Plant receptors recognize Herbivore-Associated Molecular Patterns (HAMPs), Microbe-Associated Molecular Patterns (MAMPs) and Damage-Associated Molecular Patterns (DAMPs) and trigger the induction of defenses (Mithöfer et al., 2005; Frost et al., 2007; Staudacher et al., 2017; Santamaria et al., 2018a). Plant responses are specific to the phytophagous pest species (Stout et al., 1998; de Vos et al., 2005; Rodriguez-Saona et al., 2010) and dependent on the duration of the infestation (Kant et al., 2004). However, the perception of herbivory is not well understood and few plant receptors have been identified (Bonaventure, 2012; Santamaria et al., 2018a).

Among plant defenses, protease inhibitors (PIs) exert direct effects on herbivores by interfering with their physiology (Diaz and Santamaria, 2012; Martinez et al., 2016). When an arthropod ingests PIs in its diet, the inhibition of proteolytic activities takes place in its gut avoiding the degradation of proteins. In this context, PI classification may be based on the type of protease they inhibit. There are three main subclasses of proteases involved in arthropod digestion, serine-, cysteine-, and aspartic-proteases, grouped according to the reactive amino acid of their active site group (Terra and Ferreira, 2012). The proteolytic activity is dependent on the pH of the gut (Ortego, 2012; Martinez et al., 2016). Most lepidopteran, orthopteran and hymenopteran and some coleopteran possess alkaline midguts and their digestive systems are largely based on serine-proteases and exopeptidases (Wolfson and Murdock, 1990; Ortego et al., 1996; Johnson and Rabosky, 2000). The majority of coleopteran, hemipteran and some phytophagous acari have slightly acidic midguts providing cysteine- and aspartic-proteases and exopeptidases their major proteolytic activity (Murdock et al., 1987; Cristofoletti et al., 2003; Carrillo et al., 2011). Since Green and Ryan (1972) reported that wound-inducible PIs inhibited digestive herbivore gut proteases, numerous plant PIs have been characterized for their potential to control herbivorous insects (Hilder et al., 1987; Alfonso-Rubi et al., 2003; Tamhane et al., 2005; Telang et al., 2009; Chen et al., 2014). According to MEROPS database, there are currently 85 families of PIs (Rawlings et al., 2018), being the Kazal, Kunitz, Bowman-Birk, Potato I and II, Cystatin, Cereal trypsin/α-amylase, and Serpin families the most represented in plants (Santamaria et al., 2014). Most of them specifically inhibit a mechanistic class of proteases but some may act as multifunctional inhibitors (Grosse-Holz and van der Hoorn, 2016). The first successful PI gene used to improve resistance against larvae of *Heliothis virescens* when expressed in transgenic tobacco was the cowpea trypsin inhibitor gene (CPTI) from the Bowman–Birk family (Hilder et al., 1987). Then, the CPTI gene was inserted in the genome of other plants like cotton, rice, cabbage, strawberry, sweet potato, potato or pigeon pea enhancing the resistance to different lepidopteran species (reviewed in Diaz and Santamaria, 2012). The I3 Kunitz Trypsin Inhibitor (KTI) gene family is a complex family composed by versatile protease inhibitors. Most of them inhibit serine proteases (families S1 and S8), but some of them are able to inhibit cysteine proteases (families C1 and C13) as well as other hydrolases (Renko et al., 2012). This family has been studied in different plants and contexts but most works have been focused on their potential role in defense against insect attack since their gene expression is up-regulated in response to wounding, jasmonates and insect feeding (Major and Constabel, 2008; Philippe et al., 2009; Botelho-Junior et al., 2014). *In vitro* assays with KTIs from poplar and soybean expressed as recombinant proteins differentially inhibited midgut proteases from *Mamestra configurata* and *Malacosoma disstria*, lepidopteran pests from *Populus* and crucifers, respectively (Major and Constabel, 2008). KTIs from the passion fruit displayed activity against midgut serine and cysteine proteases from the sugarcane borer *Diatraea saccharalis* and the coleopteran *Callosobruchus maculatus* on artificial diets (Botelho-Junior et al., 2014). In addition, the heterologous expression of a good number of KTIs in poplar, sweet corn, potato, rice, tobacco, and tomato conferred resistance to lepidopteran (Confalonieri et al., 1998; Cipriani et al., 1999; Gatehouse et al., 1999; Lee et al., 1999; McManus et al., 1999; Nandi et al., 1999; Marchetti et al., 2000; Rufino et al., 2013; Guimarães et al., 2015), coleopteran (Major and Constabel, 2008) and acari (Castagnoli et al., 2003). Likewise, serine-PIs from other different families overexpressed in several plant species have conferred resistance to lepidopteran, coleopteran, homopteran (reviewed in Diaz and Santamaria, 2012) and acari (Santamaria et al., 2012).

*Tetranychus urticae* is an extreme polyphagous pest with more than 1,100 documented host plants and an extraordinary ability to develop pesticide resistance (Van Leeuwen and Dermauw, 2016). These features, along with the predicted expansion of spider mites under climate change conditions, make *T. urticae* one of the most significant pests in the agriculture (Luedeling et al., 2011). Phytophagous mites pierce parenchymatic plant cells using stylets to suck their nutrients, and cause severe chlorosis leading to a reduction in crop yield (Park and Lee, 2002; Farouk and Osman, 2011; Bensoussan et al., 2016). *T. urticae* is a model within chelicerate herbivores with its genome sequenced and a broad range of tools and protocols developed (Grbic et al., 2011; Cazaux et al., 2014; Suzuki et al., 2017). Besides, mite ability to feed on *Arabidopsis thaliana* and the wide available toolkits for this plant species have provided an outstanding opportunity for functional studies of plant-mite interaction (Santamaria et al., 2012, 2015a, 2017; Zhurov et al., 2014). Among plant PIs, cystatins and serine-protease inhibitors have been reported to be involved in Arabidopsis defense against spider mite. According to Santamaria et al. (2012), the over-expression of barley cystatin (*Icy6* gene) and/or trypsin inhibitor (*Itr1* gene) conferred Arabidopsis resistance by producing an increase in mite mortality. In addition, members from I3 and I13 Potato Inhibitor I families are induced upon *T. urticae* infestation in tomato and Arabidopsis (Martel et al., 2015). In the case of plant cystatins, it is well known that their targets in mites are digestive cysteine-proteases (Carrillo et al., 2011; Santamaria et al., 2012, 2015b, 2018b). In contrast, mite targets for serine-protease inhibitors remain unknown. The fact that an Arabidopsis KTI is able to inhibit papain-like cysteine proteases and participates in the defense against herbivorous arthropods (Boex-Fontvieille et al., 2016; Rustgi et al., 2017) prompted us to examine the role of the Arabidopsis KTI protease inhibitor family in plant defense against mites. Among the seven KTIs identified in Arabidopsis, AtKTI4 and AtKTI5 were selected because of the induction of their corresponding genes after spider mite feeding, and the differences in their amino acid sequences suggesting tridimensional structure dissimilarities. Knock down lines for these two Arabidopsis *KTIs* genes were used to analyze plant phenotypes after spider mite infestation. Behavior of mites fed on knock down lines was also evaluated to verify KTI effect on mite performance. Furthermore, transient overexpression of these inhibitors in *Nicotiana benthamiana* was performed to test their ability to inhibit both serine- and cysteine-proteases.

## Materials and Methods

### Plant Material and Growth Conditions

*Arabidopsis thaliana* Columbia (Col-0), Kondara (Kon) and Bla-2 (Bla-2) accessions (Nottingham Arabidopsis Seed Collection) were used as wild-types (WT). *A. thaliana* T-DNA mutants (SALK_131716C, SALK_067224, SALK_115805C and SALK_009101C, referred as *kti4.1*, *kti4.2, kti5.1*, and *kti5.2*, respectively) were obtained from the Arabidopsis Biological Resource Centre, through the European Arabidopsis Stock Centre. For soil growth, a mixture of peat moss and vermiculite (2:1 v/v) was used. Sterilized seeds were stratified in the dark at 4°C for 5 days. Plants were then grown in growth chambers (Sanyo MLR-350-H) under control conditions (23°C ± 1°C, >70% relative humidity and a 16 h/8 h day/night photoperiod).

### Spider Mite Maintenance

A colony of *T. urticae*, London strain (Acari: Tetranychidae), provided by Dr. Miodrag Grbic (UWO, Canada), was reared on beans (*Phaseolus vulgaris*) and maintained on growth chambers (Sanyo MLR-350-H, Sanyo, Japan) at 25°C ± 1°C, >70% relative humidity and a 16 h/8 h day/night photoperiod.

### Sequence Analysis and Molecular Modeling

Multiple protein alignment was performed by MUSCLE program (Edgar, 2004). Arabidopsis Kunitz proteins were named following Santamaria et al. (2014) changing the Kun family term for the more commonly used KTI term. The KTI of *Delonix regia* (PDB ID 1R8N) was included in the alignment to infer secondary structure locations. Displayed multiple sequence alignment was made by the ESPript 3.0 web server (Robert and Gouet, 2014). 3D modeling was performed using SWISS-MODEL online protein structure prediction tool (Biasini et al., 2014). The known structures of two KTIs (PDB IDs: 3I2A and 3IIR) were used to construct the models for AtKTI4 (At1g73260) and AtKTI5 (At1g17860), respectively. Predictions on papain–AtKTI4 and papain–AtKTI5 interactions were made by using the obtained 3D models and the 3D structure of papain (PDB ID: 1PPN) in the ClusPro 2.0 server (Kozakov et al., 2017). Molecular models were visualized and analyzed by Chimera 1.12 program (Pettersen et al., 2004).

### Nucleic Acid Analysis

Genomic DNA was isolated from Arabidopsis T-DNA insertion and control lines essentially as described by Sambrook and Russell (2001). The presence and homozygous status of the T-DNA insertion lines were validated by conventional PCR (Bio-Rad) (Supplementary Figure S1). Specific primers were designed through the Salk Institute website^1^. Primer sequences are indicated in Supplementary Table S1.

For quantitative real time PCR (RT-qPCR) studies, Arabidopsis rosettes from T-DNA insertion and control lines were collected, frozen into liquid N_2_ and stored at –80°C until used for RNA isolation. Total RNA was extracted by the phenol/chloroform method, followed by precipitation with 8 M LiCl (Oñate-Sánchez and Vicente-Carbajosa, 2008). Regarding *N. benthamiana* assays, total RNA was extracted from plants agroinfiltrated with 35S::GFP, 35S::KTI4-GFP and 35S::KTI5-GFP by the TRIZOL reagent following manufacturer instructions (Ambion, Austin, TX, United States). Complementary DNAs (cDNAs) were synthesized from 2 μg of RNA using the Revert Aid^TM^ H Minus First Strand cDNA Synthesis Kit (Fermentas) following manufacturer’s instructions. The RT-qPCR conditions used were 40 cycles with 15 s at 95°C, 1 min at 60°C and 5 s at 65°C using FastStart Universal SYBR Green Master (Rox) (Roche). RT-qPCR was performed for three samples coming from three independent experiments as previously described (Santamaria et al., 2017) using a SYBR Green Detection System (Roche) and the CFX Manager Software 2.0 (Bio-Rad). mRNA quantification was expressed as relative expression levels (2^-dCt^) or fold change (2^-ddct^) normalized to ubiquitin or actin for *Arabidopsis* and *Nicotiana* samples, respectively (Livak and Schmittgen, 2001). Specific primers were designed through PRIMER3^2^. Primer sequences are indicated in Supplementary Table S1.

### Enzymatic Assays

Total protein extracts from the T-DNA insertion lines and control Arabidopsis rosettes were resuspended in 50 mM sodium phosphate pH 6.0, 0.15 M NaCl 2 mM EDTA, for 1 h at 4°C and treated as described in Santamaria et al. (2012). Total protein content was determined according to the method of Bradford (1976). Cathepsin B- and L-like activities were assayed using *N*-carbobenzoxy-Arg-Arg-7-amido-4-methylcoumarin (Z-RR-AMC) and *N*-carbobenzoxy-Phe-Arg-AMC (Z-FR-AMC) commercial substrates, respectively. Trypsin- and chymotrypsin-like activities were analyzed using Z-L-Arg-AMC (ZLA-AMC) and Suc-Ala-Ala-Pro-Phe-AMC (Suc-A-A-P-F-AMC) commercial substrates, respectively.

Inhibitory activity of plant protein extracts was tested *in vitro* against commercial trypsin (EC 3.4.21.4), chymotrypsin (EC 3.4.21.1), papain (EC 3.4.22.2), and bovine cathepsin B (EC 3.4.22.1) from Sigma. Basically, 20 μg of protein extracts were preincubated for 10 min with 100 ng of cathepsin L- and B-like in a buffer containing 100 mM sodium phosphate pH 6.0, L-cysteine, 10 mM EDTA, and 0.01% (v/v) Brij35, or with 100 ng of trypsin/chymotrypsin in the buffer 0.1 M Tris-HCl pH 7.5. Subsequently, substrates were added at a final concentration of 25 μM and incubated for 1 h at 28°C or 37°C for cysteine and serine proteases, respectively. Fluorescence was measured using an excitation filter of 365 nm and an emission filter of 465 nm (Tecan GeniusPro). The system was calibrated with known amounts of 7-amido-4-methylcoumarin (AMC) hydrolysis product in a standard reaction mixture. Specific enzymatic activity was represented as nmoles of substrate hydrolyzed/min/mg of protease. Inhibitory activity was expressed as percentage of protease activity relative to that in the absence of the inhibitor. All assays were carried out in triplicates and blanks were used to account for spontaneous breakdown of substrates.

### Subcellular Location

To create the translational fusions of At*KTI4* and At*KTI5* genes to the Green Fluorescent Protein (*GFP*) reporter gene, the corresponding cDNAs were amplified by conventional PCR using specific primers (Supplementary Table S1). The amplicons were independently cloned in-frame with the *GFP* gene into the Gateway binary vector pGWB5 (Invitrogen), under the CaMV35S promoter. 35S-Red Fluorescent Protein (RFP)-HDEL plasmid was used as a control of ER location (Shockey et al., 2006). Transient transformation of onion (*Allium cepa*) epidermal cells was performed by particle bombardment with a biolistic helium gun device (DuPont PDS-1000; Bio-Rad) as described by Diaz et al. (2005). Fluorescent images were acquired after 24 h of incubation at 22°C in the dark, using a Leica TCS-SP8 confocal microscope. GFP and RFP signals were acquired sequentially using the following settings: GFP, excitation 488 nm and emission 492–552 nm; RFP, excitation 561 nm, emission 581–665 nm.

For *N. benthamiana* agroinfiltration, the *Agrobacterium tumefaciens* strain C58CI Rif^R^ (GV3101) carrying the constructs 35S::*KTI4*-GFP (pGWB5), 35S::*KTI5*-GFP (pGWB5) or 35S::GFP (pEAQ-HT-GFP) was co-incubated with the construct 35S::P19 (pBIN61) that carries the helper P19 (Voinnet et al., 2015) to a final optical density of 0.3 nm. Bacterial suspensions were infiltrated into the abaxial side of the third-youngest fully expanded *N. benthamiana* leaf using a syringe. Fluorescent images were acquired 3 days post-infiltration (dpi), using a Leica TCS-SP8 confocal microscope. GFP and chlorophyll autofluorescence signals were acquired sequentially using the following settings: GFP, excitation 488 nm and emission 500–600 nm; chlorophyll, excitation 633 nm, emission 639–727 nm.

### Plant Damage Determination

Damage quantification analyses were done on *A. thaliana* plants from T-DNA insertion lines and WT control. Three week-old plants were infested with 20 *T. urticae* adults per plant. After 4 days of infestation, leaf damage was assessed by scanning the entire rosette using a hp scanjet (HP Scanjet 5590 Digital Flatbed Scanner series), according to Cazaux et al. (2014). Leaf damage was calculated in mm^2^ using Adobe Photoshop CS software. Six replicates were used for each genotype. Plant damage was also evaluated by analyzing accumulation of hydrogen peroxide (H_2_O_2_) and the cell death in response to spider mite attack. Leaf disks (9 mm diameter) from 3 week-old plants from the five studied genotypes, were infested with 10 mites during 24 h. The H_2_O_2_ accumulation was analyzed using 3,3-diaminobenzidine tetrachloride hydrate (DAB) substrate which produces a brown precipitate after oxidation in the presence of H_2_O_2_ (Martinez de Ilarduya et al., 2003). The staining procedure was performed according to Rodríguez-Herva et al. (2012), and observed under a Zeiss Axiophot microscope. Damage was quantified using Image J software.

Cell death was quantified by trypan blue staining as described by Sanchez-Vallet et al. (2010). Leaves were boiled in trypan blue solution [10 ml lactic acid (Sigma)], 10 ml phenol (Sigma), 10 ml glycerol (Duchefa), 10 ml water and 10 mg trypan blue (Sigma) diluted with 96% (1:2 v/v) ethanol in a 15 ml tube for 1 min. Tissues were incubated in the staining solution overnight at room temperature. Subsequently, leaves were cleared four times with 2.5 g/ml of chloral hydrate solution (PRS). Cleared leaves were prepared for imaging in 50% (v/v) glycerol, 0.1 M sodium phosphate buffer at pH 7.0, and observed under a Zeiss Axiophot microscope. Damage was quantified using Adobe Photoshop CS6 and values relativized to control plants. Six replicates were used for each genotype.

### Spider Mite Performance

Mite bioassays were conducted on entire detached Arabidopsis leaves from the T-DNA insertion and control plants. Entire leaves were fit into a closed system with 100 eggs. Samples were maintained under controlled conditions at 25 ± 1°C, >70% relative humidity and a 16 h/8 h day/night photoperiod. To study mortality, the percentage of neonate larvae (<24 h of hatching) that became adults was recorded after 10 days of feeding. Eight replicates were used for each genotype. For fecundity assays detached entire leaves were infested with 12 synchronized females each, and the number of eggs laid was counted after 36 h. Female mite synchronization was conducted on bean leaves. Entire detached leaves were placed onto wet cotton, surrounded by wet filter paper to avoid mite escape in confined special dishes (11.5 cm diameter with ventilation). 50 female mites were placed on each leaf and removed after 36 h. After 11 days, same age females were used to infest Arabidopsis leaves for the fecundity assay.

### Inhibitory Ability of *KTIs* Overexpressed in *Nicotiana*

To test the ability of AtKTI4 and AtKTI5 to inhibit cysteine/serine proteases, we used extracts from *N. benthamiana* plants co-agroinfiltrated with 35S::KTI4-GFP (pGWB5), 35S::KTI5-GFP (pGWB5) or 35S::GFP (pGWB5), and 35S::P19 (pBIN61). Entire agroinfiltrated leaves were collected at 3 dpi (days post infiltration). Fluorescent images were acquired using a Leica Fluorescence Stereoscope MZ10F. Leaves were homogenized in a buffer containing 50 mM Tris-HCl pH 7.25, 150 mM NaCl, 2 mM EDTA and 0.1% (v/v) Triton X-100, centrifuged at 16,630 *g* at 4°C for 10 min and the supernatants were pooled to obtain soluble protein extracts for inhibition assays. The ability to inhibit commercial papain, cathepsin-B, trypsin and chymotrypsin activities was *in vitro* tested as indicated above but using 15 μg of plant protein extracts for the assays. In addition, E-64 [*trans*-Epoxysuccinyl-L-leucylamido (4-guanidino)butane], TLCK (*N*_α_-Tosyl-L-lysine chloromethyl ketone hydrochloride) and chymostatin [*N*-(*N*_α_-Carbonyl-Cpd-X-Phe-al)-Phe] inhibitors (Sigma) were added as positive inhibitory controls for papain and cathepsin-B, trypsin and chymotrypsin inhibition, at a final concentrations of 0.02, 100, and 0.1 μM, respectively, and incubated for 10 min before the addition of substrates.

### Statistical Analysis

Differences in gene expression, leaf damage, mortality and fecundity assays were compared by One-Way ANOVA, followed by Student Newman–Keuls multiple comparison test (*p* < 0.05). Compensation effects and enzymatic assays were compared by One-Way ANOVA followed by Dunnett’s multiple comparison test (*p* < 0.05).

## Results

### Gene Expression of Arabidopsis I3 Kunitz Inhibitors in Response to *T. urticae* Infestation

**FIGURE 1:**
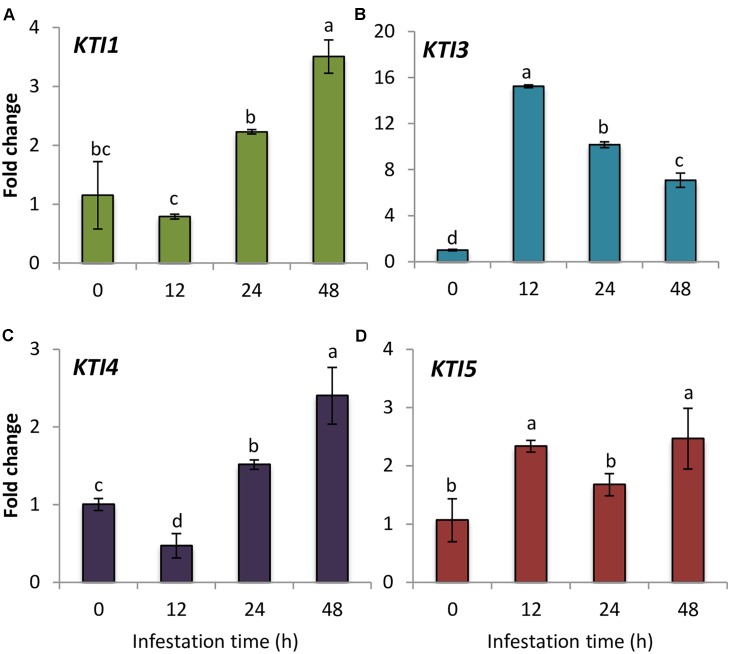
Gene expression of Arabidopsis Kunitz-type inhibitors upon spider mite infestation. Fold change in Col-0 plants at 0, 12, 24, and 48 hpi using time 0 as a calibrator sample. **(A)** At*KTI1* **(B)** At*KTI3* **(C)** At*KTI4*, and **(D)** At*KTI5*. Data are means of three replicates ± SE. Different letters indicate significant differences (*P* < 0.05, One-way ANOVA followed by Student–Newman–Keuls test).

As a first estimation of the importance of the I3 Kunitz inhibitors in Arabidopsis defense against spider mite, the gene expression of the whole Kunitz family members in infested and non-infested Col-0 plants was studied. This family included seven genes previously identified (Ma et al., 2011; Santamaria et al., 2014). One of these genes, At*KTI4* was previously reported as a differential expressed gene between the resistant Bla-2 and the susceptible Kondara Arabidopsis accessions upon spider mite feeding (Zhurov et al., 2014). RT-qPCR results showed that At*KTI5* and At*KTI3* genes were induced at 12 hpi, whereas At*KTI4* and At*KTI1* genes were up-regulated at 24 hpi, and presented their highest gene expression peak at 48 hpi (**Figure 1**). In contrast, At*KTI2*, At*KTI6*, and At*KTI7* gene expression was not detected in Arabidopsis leaves neither in non-infested plants nor upon mite infestation.

### Sequence and Structural Features of Arabidopsis KTIs

**FIGURE 2:**
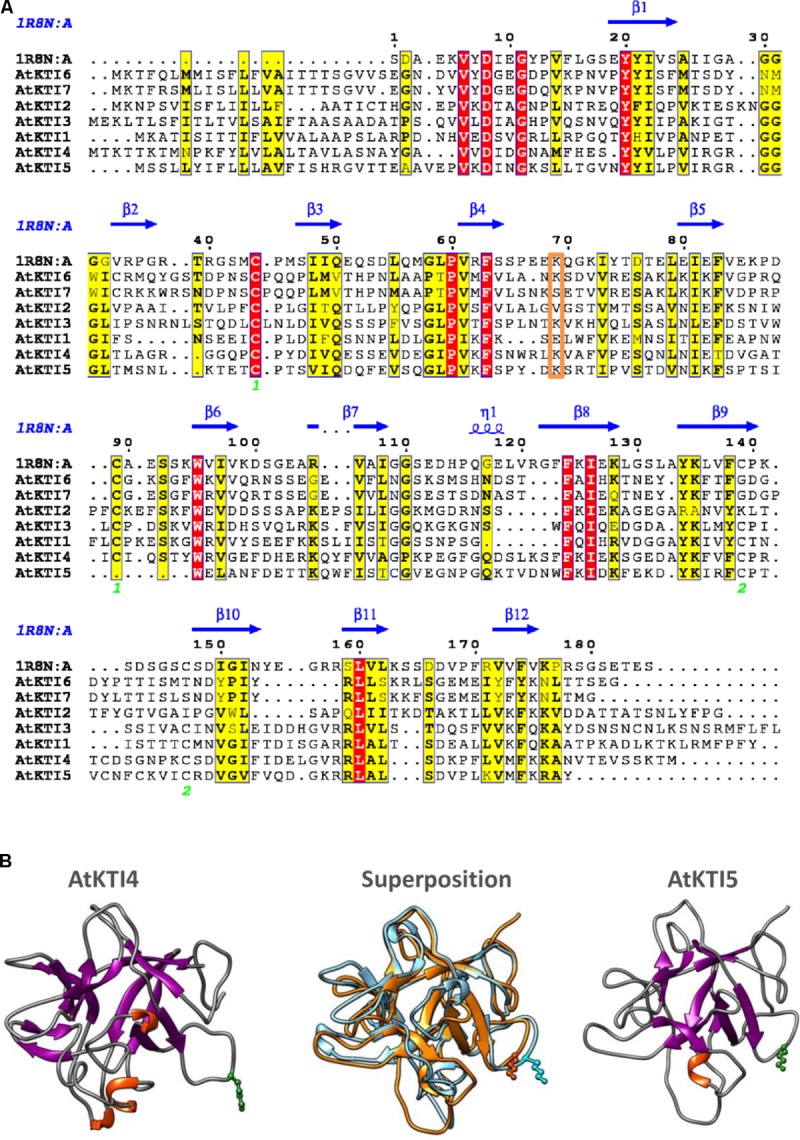
Sequence-structure analysis of Kunitz-type inhibitors. **(A)** Multiple sequence alignment of Arabidopsis KTIs. The Kunitz type inhibitor of *Delonix regia* (PDB ID 1R8N) was included in the alignment to infer secondary structure locations. Conserved residues are in red boxes. Similar residues are indicated in black bold characters and boxed in yellow. Green numbers at the bottom indicate disulphide bridge topology. Putative reactive site is boxed in orange. The figure was made with the ESPript 3.0 web server. **(B)** Ribbon diagrams showing structural models for AtKTI4 and AtKTI5, and their superposition (blue, AtKTI4; orange, AtKTI5). Conserved reactive Lys residue is colored in green. Modelization and visualization were made by SWISS-Model and Chimera tools.

To obtain some clues on the inhibitory capacities of the seven Arabidopsis KTIs, their amino acid sequences were aligned. The seven inhibitors presented a signal peptide in the N-terminal region and their sequences were more similar in the regions aligned with the amino acids involved in secondary structures of a Kunitz inhibitor from *D. regia* (**Figure 2A**). The AtKTI2 did not present the positively charged residue (Lys or Arg) in the loop between strands β4 and β5, essential to inhibit trypsin. This residue was at the right position in AtKTI3, 4, 5, and 6. The amino acid pair Trp-Pro, located in the loop between strands β5 and β6, and putatively involved in cysteine-protease inhibitory capability of AtKTI2 was only partially conserved in AtKTI1 and AtKTI3. Whereas AtKTI2, AtKTI6, and AtKTI7 lacked the two cysteines involved in the first disulphide bridge, AtKTI5 is deficient in one of them but had two additional cysteines between strands β9 and β10 that could form a novel disulphide bridge. To determine the possible effect of this variation on the protein structure, tridimensional structures for AtKTI4 and 5 were made by homology modeling. Predictions showed structural differences. This predicted structural dissimilarity was mainly observed in the loops connecting secondary structures and leads to a distinct spatial orientation of the Lys reactive residue in the β4–β5 loop (**Figure 2B**). The predicted sequence-structure plasticity of these inhibitors could lead to different inhibitory properties. Thus, AtKTI4 and 5 were selected to further characterization.

### AtKTI4 and AtKTI5 Protein Subcellular Location

**FIGURE 3:**
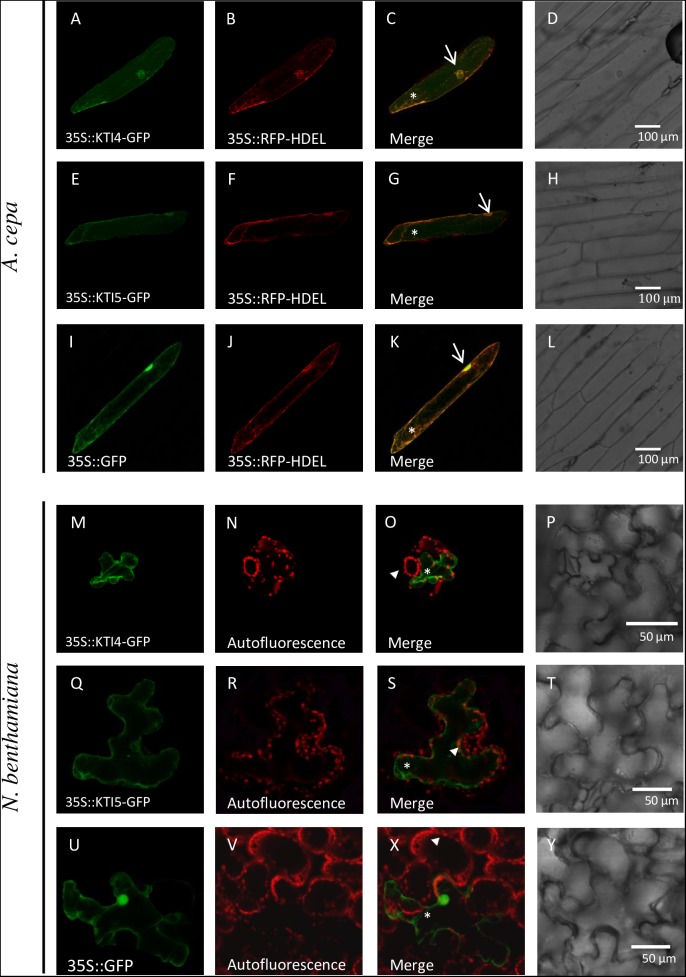
AtKTI4 and AtKTI5 subcellular localization. Confocal stacks spanning epidermal onion cells co-transformed with 35S::KTI4-GFP, 35S::KTI5-GFP and 35S::GFP and 35S::RFP-HDELcontrols. Confocal images and projections of the KTI4 **(A)** and KTI5 **(E)** are shown. Projections from GFP **(A,E,I)**, RFP **(B,F,J)**, merged **(C,G,K)** and the corresponding Nomarski snapshots **(D,H,L)**. Confocal stacks spanning *N. benthamiana* cells agroinfiltrated with 35S::KTI4-GFP **(M)**, 35S::KTI5-GFP **(Q)** and 35S::GFP control **(U)**. Projections from GFP **(M,Q,U)**, chlorophyll auto fluorescence **(N,R,V)**, merged **(O,S,X)** and the corresponding Nomarski snapshots **(P,T,Y)**. Bars are indicated in images. Arrows indicate nuclei, asterisks signal ER and arrowheads the chlorophyll autofluorescence.

Signal peptides found in every AtKTI indicate a targeted transport to the endoplasmatic reticulum. To determine the final AtKTI4 and AtKTI5 subcellular localization, transient expression assays were performed in onion epidermal layers by microparticle bombardment. AtKTI4 and AtKTI5 were fused to GFP. To determine the subcellular localization of the GFP signal, the cells were co-transfected with the 35S::RFP-HDEL plasmid to reveal the ER. Both green and red signals were found in the nuclear area, tracking along threads of cytoskeleton elements and the cell periphery. These locations are consistent with the subcellular distribution of the ER (**Figures 3A–C,E–G**). The 35S::GFP control showed an intense fluorescence in the cell nucleus, entering due to its small size, whereas the ER marker is seen in the nuclear periphery (**Figures 3I,J**). Agroinfiltration of *N. benthamiana* plants with the same constructs confirmed the location of both AtKTI4 and AtKTI5 inhibitors in the endomembrane system. In this case, the red signal corresponds to the autofluorescence of chlorophyll (**Figures 3M–V,X,Y**).

### Effects of Knock-Down AtKTI4 and AtKTI5 Lines on KTI Expression and Protease Inhibitory Properties

To investigate the role of AtKTI4 and AtKTI5 proteins in plant defense, silenced lines for these genes (*kti4.1, kti4.2*, *kti5.1*, *kti5.2*) were ordered. The characterization of the homozygous mutant lines revealed the insertion of the T-DNA at the promotor region for *kti4.1* and *kti5.2* lines, at the coding region for *kti5.1* line and at the 3^′^UTR for the *kti4.2* line (Supplementary Figure S1A).

**FIGURE 4:**
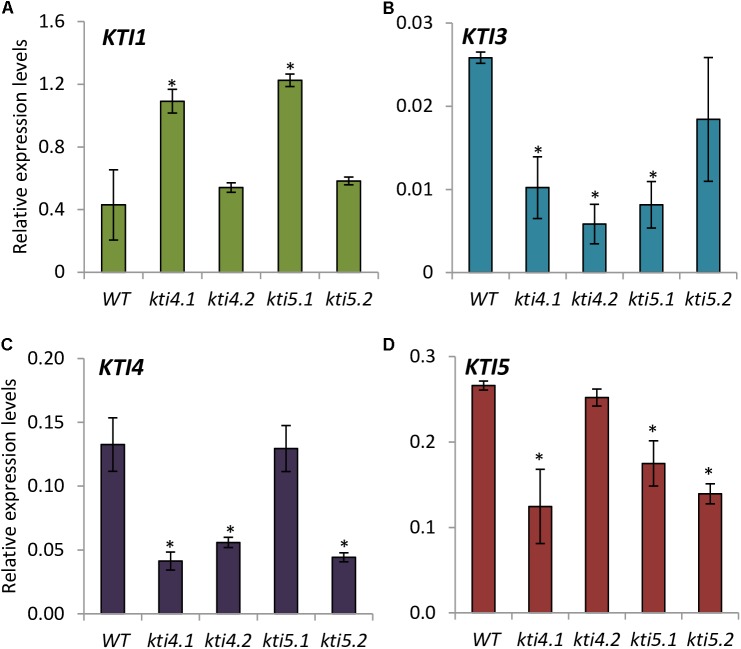
Gene expression of Kunitz-type inhibitors in Arabidopsis T-DNA insertion lines and WT plants. Relative gene expression levels of: **(A)** At*KTI1* **(B)** At*KTI3* **(C)** At*KTI4* and **(D)** At*KTI5*. Data are means ± SE of three replicates. Asterisk indicates significant differences with the WT (*P* < 0.05, One-way ANOVA followed by Dunnett’s test).

The expression of At*KTI1*, At*KTI3*, At*KTI4*, and At*KTI5* genes was analyzed in the *kti4* and *kti5* mutants and in the WT plants. As expected, the expression of At*KTI4* and At*KTI5* genes was reduced in their cognate T-DNA insertion lines (**Figures 4C,D**). Moreover, statistical differences in mRNA quantification revealed variations in the expression of other At*KTI* genes in these lines. Regarding the mutant lines for At*KTI4* gene, *kti4.1* plants were knocked down for At*KTI3*, At*KTI4*, and At*KTI5* genes and up regulated for At*KTI1* gene (**Figures 4A–D**) while *kti4.2* plants were down regulated for At*KTI3 and* At*KTI4* genes (**Figure 4C**). In the case of the T-DNA insertion lines for At*KTI5* gene, *kti5.1* plants were knocked down for At*KTI3* and At*KTI5* genes while the expression of At*KTI1* gene was induced (**Figures 4A,B,D**), and *kti5.2* mutant plants were down regulated for At*KTI4* and At*KTI5* genes (**Figures 4B–D**).

**FIGURE 5:**
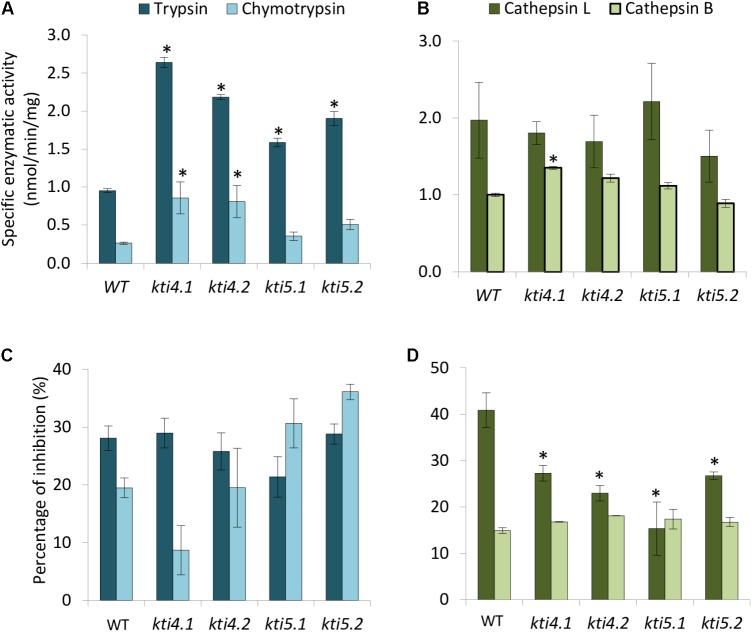
Proteolytic patterns of T-DNA insertion lines. Specific proteolytic activities of protein extracts from Arabidopsis T-DNA insertion lines and control WT using specific substrates. **(A)** Trypsin- and chymotrypsin-like specific activities. **(B)** Cathepsin L- and B-like specific activities. Data are expressed as nmoles/min/mg. Inhibitory activity of protein extracts from T-DNA insertion lines and control plants against commercial proteases. **(C)** Inhibitory activity against trypsin and chymotrypsin. **(D)** Inhibitory activity against commercial papain (cathepsin L-like) and bovine cathepsin B. Data are expressed as a percentage of inhibition. Data are means ± SE of three replicates. Asterisk indicates significant differences with the WT (*P* < 0.05, One-way ANOVA followed by Dunnett’s test).

Compensatory effects described above were also studied by analyzing the effect of T-DNA insertions on the protease activities of these plants. Results showed higher trypsin activity in all mutants than in the WT plants (**Figure 5A**). For chymotrypsin activity, *kti4.1* and *kti4.2* plants presented higher proteolytic levels in comparison to WT plants while *kti5.1* and *kti5.2* did not (**Figure 5A**). In contrast, no significant differences on both, cathepsin L- and B-like activities were detected between mutant and WT plants. Only the *kti4.1* line showed slightly higher levels of cathepsin B-like activity than the WT plants (**Figure 5B**). The capability of the different Arabidopsis genotypes to inhibit serine- and cysteine-proteases was also tested by using commercial proteases. Significant differences were not found for trypsin, chymotrypsin and bovine cathepsin B assays (**Figures 5C,D**). Interestingly, the commercial papain (cathepsin L-like) was less inhibited by protein extracts from mutant lines than from the WT plants (**Figure 5D**).

### AtKTI4 and AtKTI5 Are Able to Inhibit Both Serine and Cysteine Proteases

**FIGURE 6:**
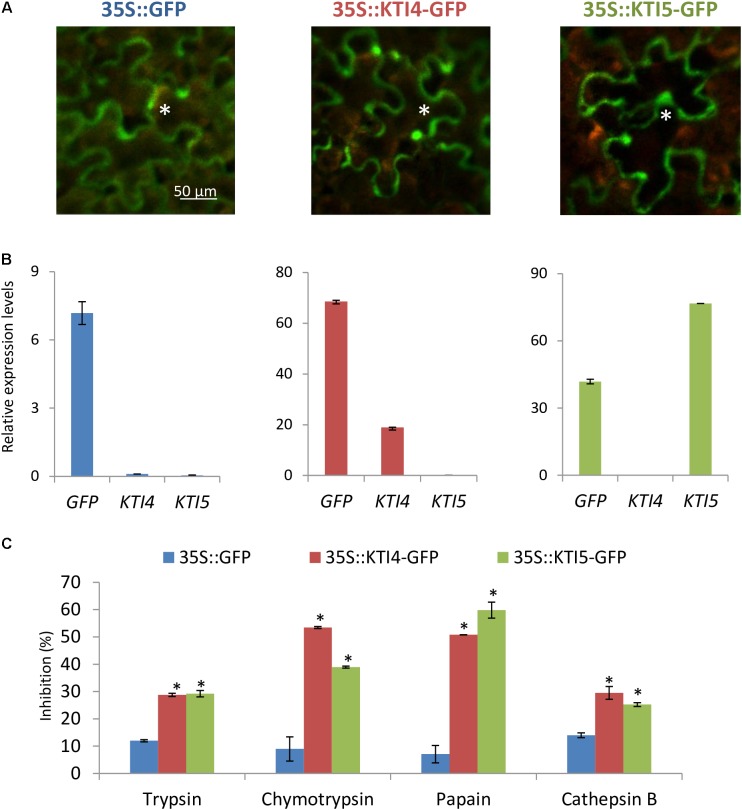
Characterization of the expression, location and inhibitory properties of At*KTI4* and At*KTI5* genes by using transient expression assays in *N. benthamian*a plants. **(A)** GFP signal after 3 days of agroinfiltration. Asterisks indicate GFP signal of the epidermal cells. **(B)** Relative expression levels of *GFP*, *KTI4*, and *KTI5* genes in the *N. benthamiana* plants after 3 days of infiltration. **(C)** Inhibitory ability of *N. benthamiana* protein extracts expressing *KTI4* and *KTI5* genes against commercial trypsin, chymotrypsin, papain and bovine cathepsin B 3 days post-agroinfiltration. Data are mean ± SE of triplicate measurements of each sample. Asterisk indicates significant differences with the 35S::GFP plants (*P* < 0.05, One-way ANOVA followed by Dunnett’s test).

To further explore the serine- and cysteine-protease inhibitory capacity of AtKTI4 and AtKTI5 inhibitors, *Agrobacterium*-mediated transient over-expression for At*KTI4* and At*KTI5* genes fused to GFP was performed in *N. benthamiana* plants. GFP detection demonstrated the expression of the GFP gene and the location of the GFP protein in all agroinfiltrated plants (**Figures 6A,B**). Protein extracts from plants expressing the AtKTI4 and AtKTI5 proteins fused to GFP presented higher capability to inhibit serine- (trypsin and chymotrypsin) and cysteine- proteases (papain and bovine cathepsin B) compared with the extracts from the plants expressing only the GFP protein (**Figure 6C**). These results support the consideration of AtKTI4 and AtKTI5 as bifunctional inhibitors of both serine and cysteine proteases.

Docking analyses using ClusPro program were carried out to figure out the putative interaction between papain and the inhibitors AtKTI4 and AtKTI5. Models suggest an intrusion of different residues into the reactive site of papain stabilized by hydrogen bonds (Supplementary Figure S2). In the AtKTI4-papain interaction, the residues Glu122 and the Lys175 at the β6–β7 and β9–β10 loops, respectively, would form hydrogen bonds with the Cys and His amino acids of the papain reactive site. A similar interaction by the residues Val164 and Lys165 at the β9–β10 loop of AtKTI5 was predicted. Additional hydrogen bonds between residues of protease and inhibitor are predicted, which probably are involved in the preservation of the interaction (data not shown).

### Effects of At*KTI4* and At*KTI5* Genes on Plant Resistance and Mite Performance

**FIGURE 7:**
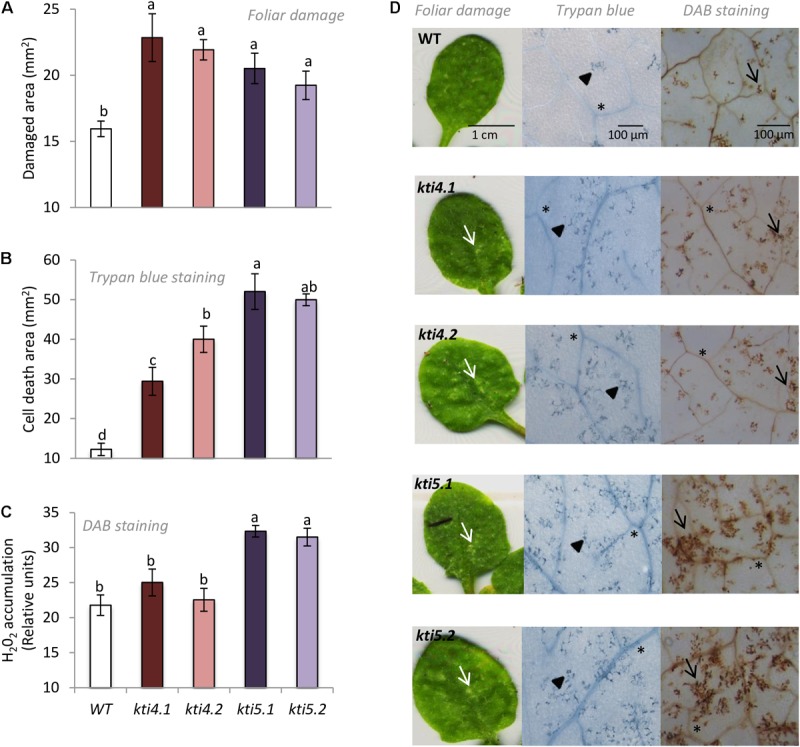
Plant damage and hydrogen peroxide production of Arabidopsis genotypes infested with *T. urticae.* **(A)** Foliar area damaged on Arabidopsis T-DNA insertion lines (*kti4.1, kti4.2, kti5.1, kti5.2*) and WT plants after 4 days of spider mite infestation. **(B)** Trypan blue stained area on leaf disks from Arabidopsis T-DNA insertion lines and WT plants after 1 day of spider mite infestation. **(C)** Hydrogen peroxide production on leaf disks from Arabidopsis T-DNA insertion lines and WT plants after 1 day of spider mite infestation. **(D)** Example of leaf phenotypes of Col-0 genotypes after spider mite infestation. Data are means ± SE of six replicates. Different letters indicate significant differences (*P* < 0.05, One-way ANOVA followed by Student–Newman–Keuls test). Bars are as indicated in images. White arrows indicate plant damage, arrowheads cell death, black arrows H_2_O_2_ accumulation and asterisks signal indicate vascular tissues.

To further explore into the role of AtKTI4 and AtKTI5 in plant defense against *T. urticae*, homozygous T-DNA insertion lines and WT plants were infested with spider mites and the plant damage (chlorotic area) was visualized and quantified 4 days upon mite infestation. All knock down lines showed more damage than the WT plants. The injury was between 1.2 and 1.5 times higher in the knock down lines than in the WT plants (**Figures 7A,D**). Cell death caused by spider mite feeding was evaluated by trypan blue staining of leaves from all Arabidopsis genotypes after 24 h of infestation. All knock down lines showed higher levels of staining than WT plants with *kti5.1* and the *kti5.2* lines being the most prominent (**Figures 7B,D**). As the production of H_2_O_2_ is used as a plant damage indicator, H_2_O_2_ concentrations were determined in the five Col-0 genotypes. The quantification of H_2_O_2_ in infested plants, expressed as DAB relative units, demonstrated that *kti5.1* and *kti5.2* lines accumulated more H_2_O_2_ than *kti4.1, kti4.2* lines and WT plants (**Figures 7C,D**).

**FIGURE 8:**
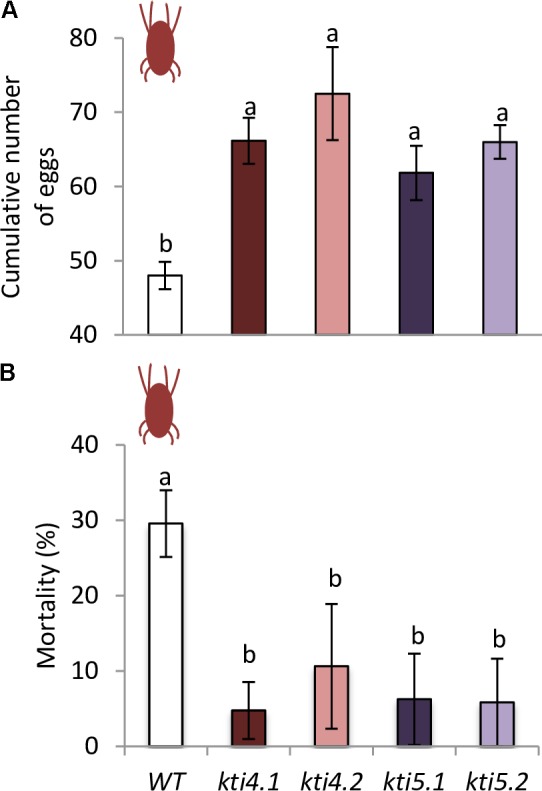
Analysis of spider mite performance. **(A)** Effects of T-DNA insertion lines and control plants on *T. urticae* fecundity, 36 h after infestation with synchronized females. **(B)** Effects of T-DNA insertion lines and control plants on *T. urticae* mortality, 10 days after infestation with neonate larvae. Data are means ± SE of eight replicates. Different letters indicate significant differences (*P* < 0.05, One-way ANOVA followed by Student–Newman–Keuls test).

To ensure that the chlorotic area correlated with mite feeding, mite performance was analyzed after feeding on mutants for At*KTI4* and At*KTI5* lines. Fecundity assays carried out on leaves from different Col-0 genotypes showed that synchronized mites fed on insertion lines had higher fecundity rates than the ones fed on WT plants (**Figure 8A**). To evaluate the KTI toxicity for mites, the mortality was recorded after feeding. Mites fed on mutant lines exhibited lower mortality rates than those on the WT plants (**Figure 8B**).

## Discussion

PIs from plants are proteins of particular interest because of their putative involvement in the natural defense system to phytophagous pests and pathogens. Particularly, the defense role of the plant KTI family against insects has been proven, although most studies involved individual Kunitz-type members. Determining the functional diversity of all members of the At*KTI* gene family against an important pest such as the polyphagous mite *T. urticae* may provide alternatives to spider mite control. From the 7 At*KTI* genes identified in Arabidopsis, four of them, At*KTI1*, At*KTI3*, At*KTI4*, and At*KTI 5* responded to spider mite feeding (**Figure 1**). At*KTI2*, At*KTI6*, and At*KTI7* genes were not expressed in Arabidopsis rosettes either under control or infested conditions. In the case of the At*KTI2*, this result was in agreement with a recent report showing a restricted expression to flowers and etiolated seedlings (Boex-Fontvieille et al., 2016).

Generally, KTIs are small proteins of about 20 kDa folded in β-trefoil manner with two disulphide bonds, but small differences in sequence or structure may make them different in their role as inhibitors of serine- and/or cysteine-proteases (Bendre et al., 2018). The low similarity observed among the amino acids residues of the 7 AtKTI members when their sequences were aligned may justify specific inhibitory roles against mite proteases (**Figure 2**). Trypsin inhibition is expected as the S1-binding site is negatively charged and accepts the conserved Lys at the P1 position. Likewise, chymotrypsin inhibition could be due to the presence of Tyr, Leu, and Phe residues in the β4–β5 reactive loop. The ability to inhibit papain could not be *a priori* predicted from the analysis of the amino acid sequences due to the multiple ways that Kunitz and Kunitz-structurally related inhibitors follow to inhibit C1 cysteine-proteases (Renko et al., 2012). These inhibitors may use different loops to occlude the reactive site of the cysteine protease. For instance, residues Trp88 and Pro89 of the AtKTI2 β5–β6 loop have been proposed to intrude into the catalytic triad of the Arabidopsis cysteine-protease RD21, blocking its protease activity (Boex-Fontvieille et al., 2015). This interaction is stabilized by hydrogen bonds between RD21 amino acids and residues in the AtKTI2 β2–β3 and β5–β6 loops. The comparative alignment of amino acid sequences of the AtKTI members revealed that AtKTI2, AtKTI6, and AtKTI7 were the most different among the whole family. These inhibitors presented the cysteine residues essential to form only one disulphide bridge instead of the two bridges predicted in the rest of AtKTIs. Besides, the AtKTI2 lacked the positive residue in the loop between strands β4 and β5 essential for trypsin inhibition, which correlated with its inability to inhibit serine-proteases. In contrast, although several dissimilarities were found in the tridimensional structures of AtKTI4 and AtKTI5, both proteins conserved the Lys residue in the β4–β5 loop and are putatively able to interact with the catalytic site of papain. This plasticity of the loops coming out of the stable β-trefoil scaffold maybe the reason of the versatility of these inhibitors, which display several different mechanisms of inhibition involving different positions of the loops and their combinations (Renko et al., 2012). Thus, the wide sequence-structure variability of KTIs supports the bifunctional action shown for AtKTI4 and AtKTI5 against trypsin and chymotrypsin serine-proteases as well as against cathepsin B- and L-like cysteine-proteases. Many protease inhibitors have to be targeted to the endomembrane system to reach their functional location (Martinez et al., 2009). As expected for proteins with signal peptide that would end up as vesicle-secreted proteins, AtKTI4 and AtKTI5 were subcellular located in the endomembrane system (**Figure 3**). Previous reports have detected AtKTI2 in the cell wall, apoplast spaces and tonoplast (Boex-Fontvieille et al., 2015).

To elucidate the response of At*KTI4* and At*KTI*5 genes after mite feeding, we studied their effect on the spider mite performance using T-DNA insertion lines for both genes. First, we confirmed that the reduction of At*KTI4* and At*KTI5* transcripts detected in the mutant lines was associated to an increase of commercial trypsin activity, detected by *in vitro* assays using plant extracts, a promising result to perform bioassays (**Figure 5**). Besides, we found compensation effects in T-DNA insertion lines through the alteration of the expression of other At*KTI* genes, suggesting that AtKTI1, AtKTI3, AtKTI4, and AtKTI5 proteins might participate in the defense process against spider mites in a concerted manner to modulate protease target activities (**Figure 4**).

Feeding assays conducted with the spider mite resulted in a significant increase of leaf damage either quantified as total chlorotic area or detected by trypan blue staining in comparison to control plants (**Figure 7**). However, only the knock down lines for the At*KTI5* gene accumulated higher levels of H_2_O_2_ than the WT plants. These findings indicate that these mutant lines either produced more H_2_O_2_ when infested, or alternatively, were not able to detoxify H_2_O_2_ efficiently, and triggered the cell death. Santamaria et al. (2017) demonstrated that an increase in H_2_O_2_ during *T. urticae* feeding was associated to sharp reduction in the accumulation of thiol groups and a parallel promotion of cell death. It is generally accepted that moderate levels of reactive oxygen and/or nitrogen species may differentially sense defense signaling while an excess of oxidative stress results to programmed cell death (Foyer and Noctor, 2005; Baxter et al., 2014). Therefore, these changes in the redox status have a potential impact on mite behavior. Accordingly, mite performance improved when fed on knock down lines (**Figure 8**). Our results confirm a significant reduction in mite mortality and higher fecundity rates upon feeding on mutant lines compared to control plants. Previous literature indicated that the role of At*KTI4* and At*KTI5* in Arabidopsis defense was not specifically associated to mites. Both genes were induced by *Botrytis cinerea* treatment (Coolen et al., 2016). At*KTI4* was also triggered by pathogen-derived elicitors and antagonized pathogen-associated cell death in Arabidopsis (Li et al., 2008), which may explain the higher cell death observed in our T-DNA insertion lines after spider mite infestations. Additionally, the expression of At*KTI4* and At*KTI5* was modulated by *Pieris rapae* infestation (Coolen et al., 2016). Interestingly, the expression pattern of At*KTI4* gene showed a highly localized response to *P. brassicae* eggs (Little et al., 2007) and was also induced upon nematode infestations in Arabidopsis (Jammes et al., 2005).

A second, but probably the most important mechanism of defense mediated by the AtKTIs is based on their capability to inhibit mite protease activities. The sequence and annotation of *T. urticae* genome revealed a large proliferation of serine- and cysteine-protease gene families in comparison to other sequenced arthropod species (Grbic et al., 2011). The 70 gene members identified in the serine-protease family pointed out an essential role in the spider mite physiology (Grbic et al., 2011). In many phytophagous insects, particularly in lepidopteran, the participation of serine proteases in the gut digestion has been demonstrated (Lara et al., 2000; Patankar et al., 2001; Fan and Wu, 2005; Srinivasan et al., 2006; Chougule et al., 2008; Tabatabaei et al., 2011). However, the lack of detection of trypsin- and chymotrypsin activities in mite extracts (Carrillo et al., 2011) together with the fact that serine-protease genes did not show a clear developmental pattern of expression correlated with feeding stages (Santamaria et al., 2012, 2015b), and the absence of this activity in mite feces suggested the association of this protease type with physiological processes other than the hydrolysis of dietary proteins (Santamaria et al., 2015b). Jonckheere et al. (2016) found some genes that presumably code for serine-proteases expressed in the salivary glands of *T. urticae* suggesting a pre-digestive function in the saliva. Based on these data, an alternative target for KTIs could be the serine-proteases present in the mite saliva. However, other putative roles involved in the regulation of mite growth and development have been suggested for these enzymes (Santamaria et al., 2012). In any case, plant inhibitors might get access to the endogenous proteases through the mite gut, as has been described for some insects (Down et al., 1999; Azzouz et al., 2005). The induction of members of the PIN-I and PIN-II serine PI families after spider mite attack (Li et al., 2002; Kant et al., 2004, 2008; Martel et al., 2015) and the enhanced resistance to spider mites showed by Arabidopsis when overexpress a barley trypsin inhibitor (Carrillo et al., 2011; Santamaria et al., 2012) strongly support the potential defense role of KTIs. Interestingly, the transient expression of At*KTI4* and At*KTI5* genes in *Nicotiana* plants showed their bifunctional features to inhibit cysteine- and serine-protease activities (**Figure 6**), which it should substantiate the impact of AtKTIs on cysteine-proteases from the mite gut involved in the hydrolysis of dietary proteins.

## Conclusion

Our results confirm a wide role of the Arabidopsis KTI proteins in defense against spider mite based on: (i) four out of the seven *KTIs* identified in *A. thaliana* are induced upon spider mite infestation; (ii) transcriptional *KTI* compensation effects take place among the silencing At*KTI4* and At*KTI5* lines; (iii) *T. urticae* inflicts more leaf damage in *kti4* and *kti5* mutant lines than in WT plants; (iv) mites feeding on At*KTI* silencing lines improve their performance; (v) AtKTI4 and AtKTI5 show a bifunctional inhibitory activity against both serine and cysteine proteases. In consequence, the inhibition of proteolytic process mediated by AtKTIs may decrease the mite access to essential amino acids and consequently to impair protein functions and to disrupt crucial physiological events needed by *T. urticae* performance. These effects finally increase mite mortality and reduce mite reproduction. Further research is needed to elucidate if the ability to inhibit serine protease activity contributes to the defense role of these inhibitors against spider mite.

## Author Contributions

ID and MM conceived the research. AA, LT-M, and MS performed most of the experimental research. ID, MM, PG-M, and MS participated in the design, the acquisition, analysis, or interpretation of data for the work. MS, MM, and ID wrote the manuscript. All authors contributed to final version of the manuscript.

## Conflict of Interest Statement

The authors declare that the research was conducted in the absence of any commercial or financial relationships that could be construed as a potential conflict of interest.

## Footnotes

1 http://signal.salk.edu/tdnaprimers.2.html

2 http://bioinfo.ut.ee/primer3-0.4.0/
