# Supplementary material for: Arabidopsis Kunitz Trypsin Inhibitors in Defense Against Spider Mites
Source: Front Plant Sci. 2018 Jul 10;9:986. doi: 10.3389/fpls.2018.00986 (PMC6048452; doi:10.3389/fpls.2018.00986)
Supplement: Supplementary file 1 [file Data_Sheet_1.DOCX]

Supplementary Material

Arabidopsis Kunitz Trypsin Inhibitors in Defence Against Spider Mites

Ana Arnaiz, Lucia Talavera-Mateo, Pablo Gonzalez-Melendi, Manuel Martinez, Isabel Diaz, M. Estrella Santamaria^*^.

*** Correspondence:** M.Estrella Santamaria: [me.santamaria@upm.es](mailto:me.santamaria@upm.es)


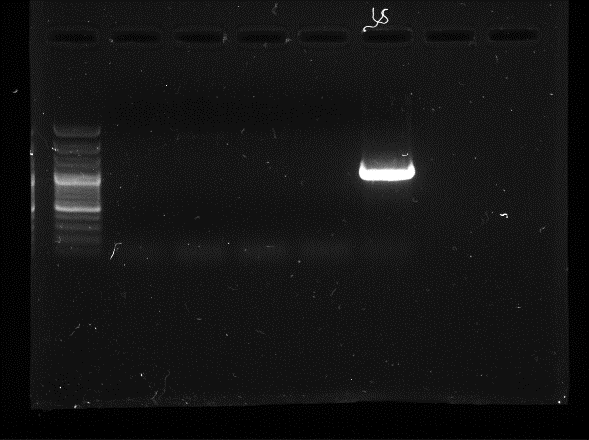

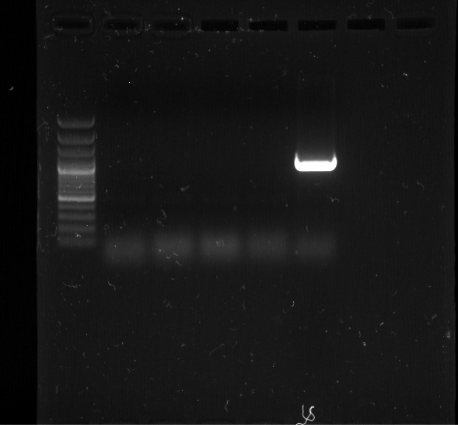

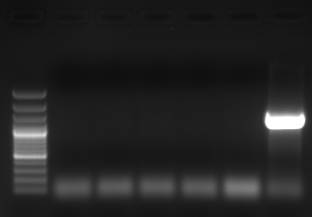


**MW 1 2 3 4 5 WT**

RP+LP


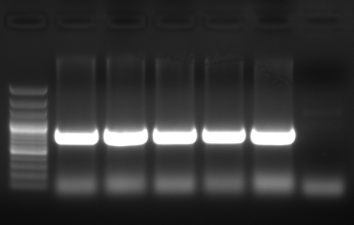


**MW 1 2 3 4 5 WT**

RP+LB

**SALK_131716C**


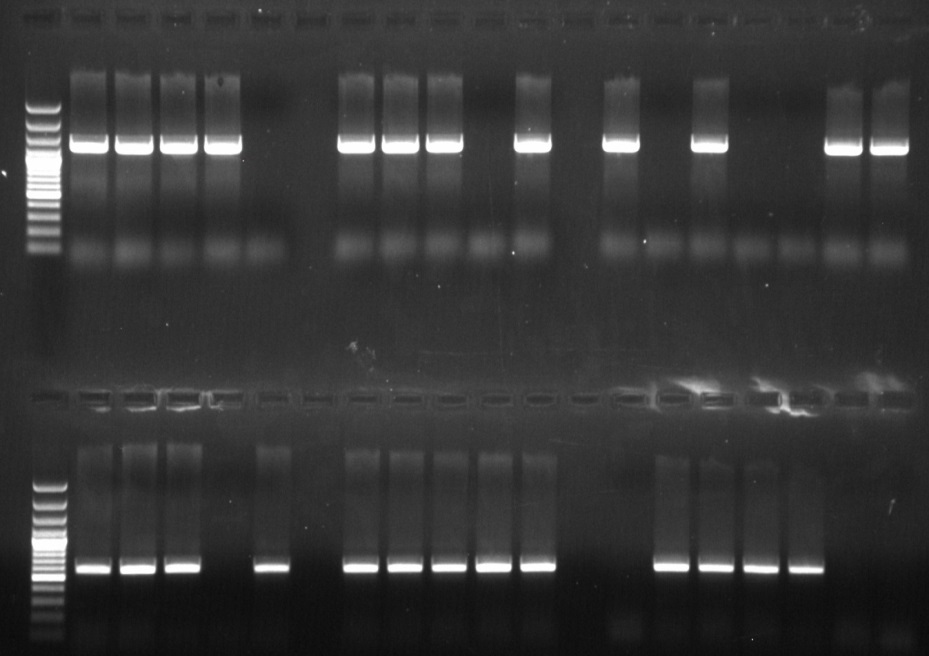

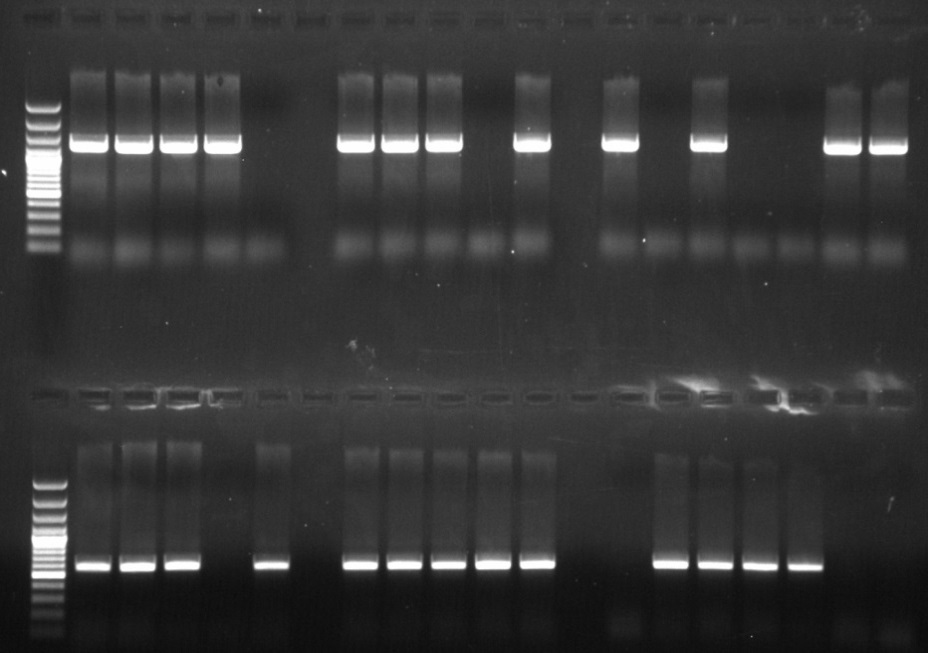


**MW 1 2 3 4 5 WT**

RP+LP


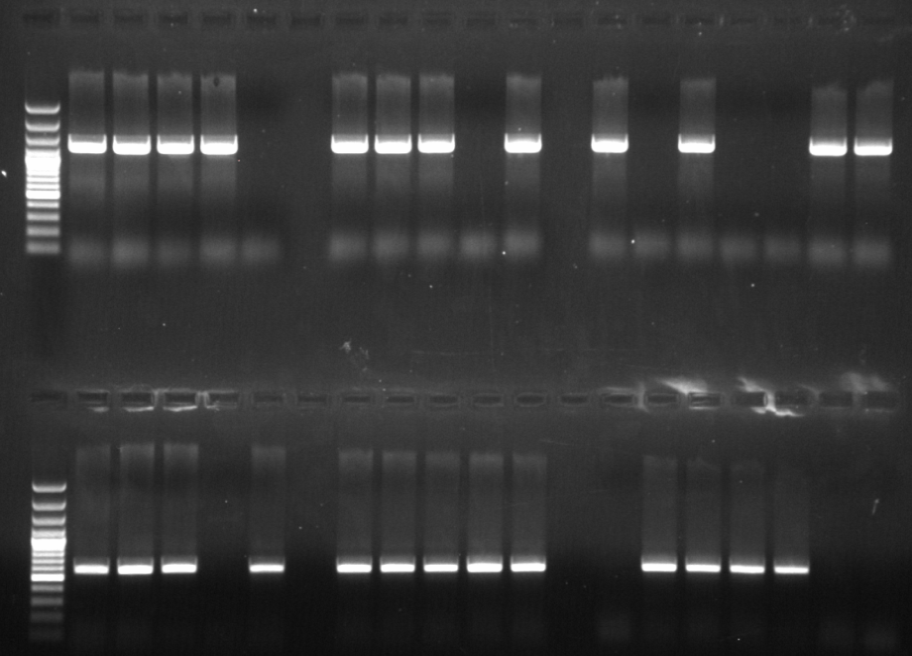

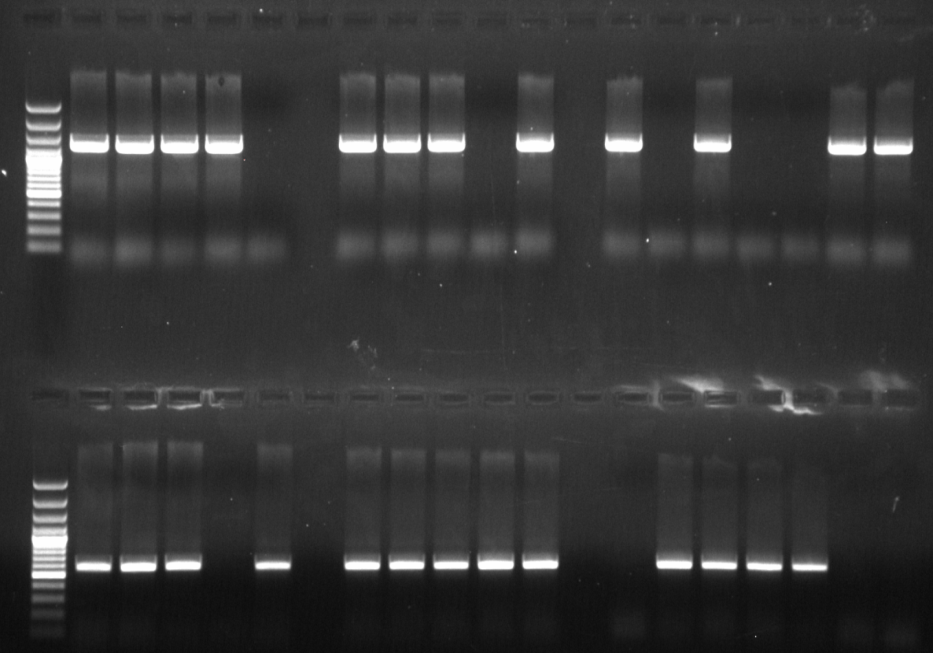


**MW 1 2 3 4 5 WT**

RP+LB

**SALK_067224 🡪 7**

**SALK_115805C**


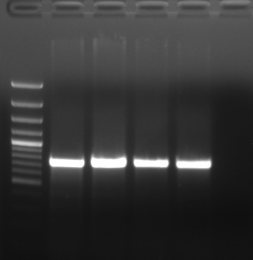


RP+LB

**MW 1 2 3 4 WT**

**SALK_009101C**

**MW 1 2 3 4 WT**


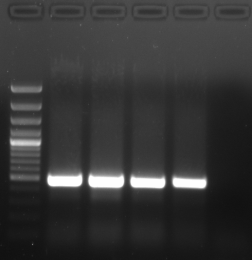


**MW 1 2 3 4 WT**

RP+LP

RP+LP

RP+LB

LP

RP

RP

T-DNA

LB

WT locus

T-DNA locus

*kti5.1* (SALK_115805C)

100 bp

*kti5.2* (SALK_009101C)

*kti4.2* (SALK_067224)

*kti4.1* (SALK_131716C)

5’ UTR

3’ UTR

Exon

3’ UTR

5’ UTR

Exon

**(A)**

**(B)**

**(D)**

**(C)**

**MW 1 2 3 4 WT**

**Supplementary Figure 1.** Molecular characterization of Arabidopsis T-DNA insertion lines. **(A)** Scheme of the position of the T-DNA insertions (arrowhead) in the mutant Salk *kti4.1* (SALK_131716C), *kti4.2* (SALK_067224), *kti5.1* (SALK_115805C) and *kti5.2* (SALK_009101C) lines. **(B)** Location of the primers used for *kti4.1*, *kti4.2*, *kti5.1* and *kti5.2* lines validation. Primer sequences are indicated in Supplementary Table 1. **(C)** PCR assays of *KTI4* gene in mutant and control lines to show homozygous status. **(D)** PCR assays of *KTI5* gene in mutant and control lines to show homozygous status.


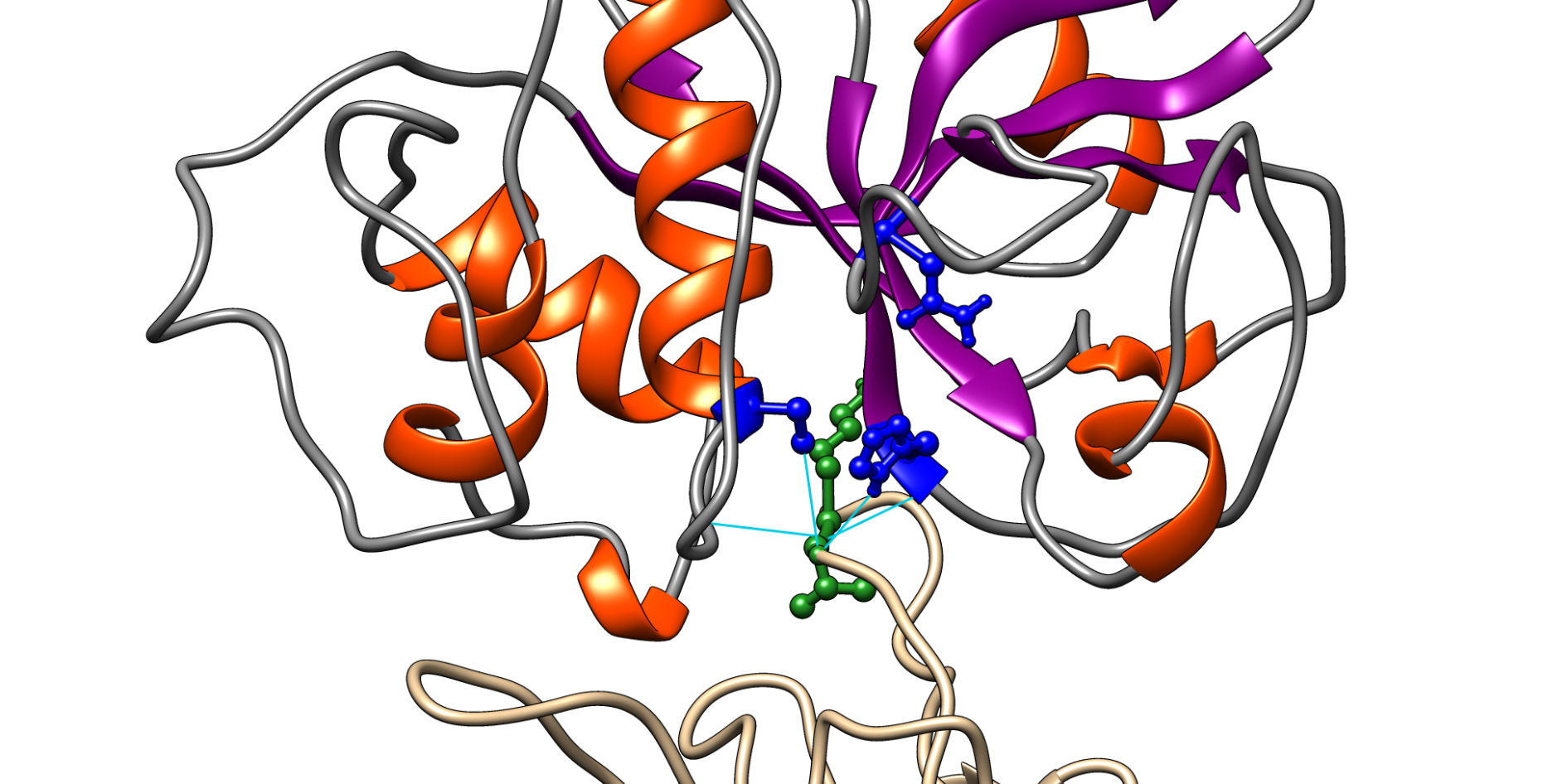

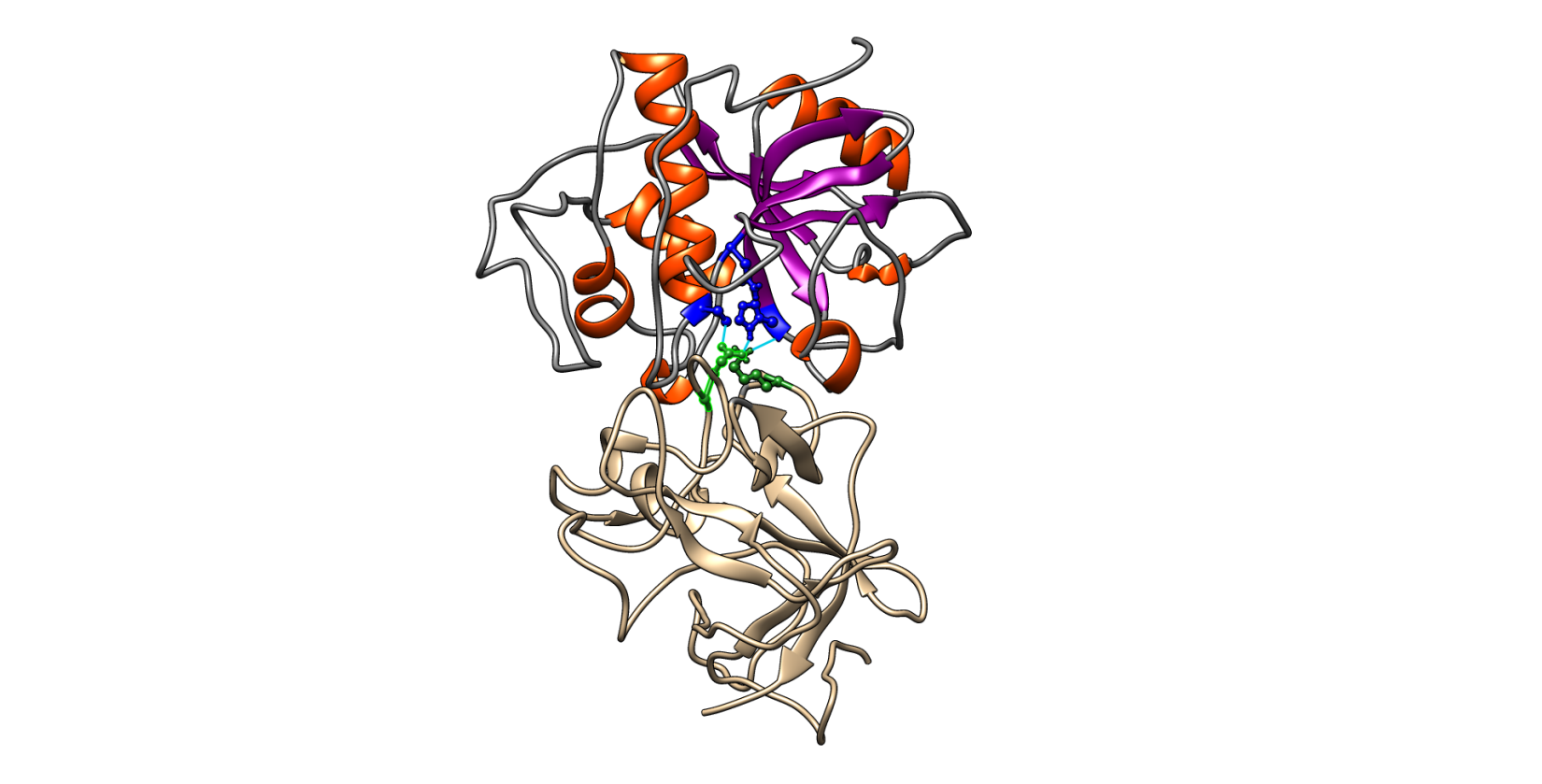

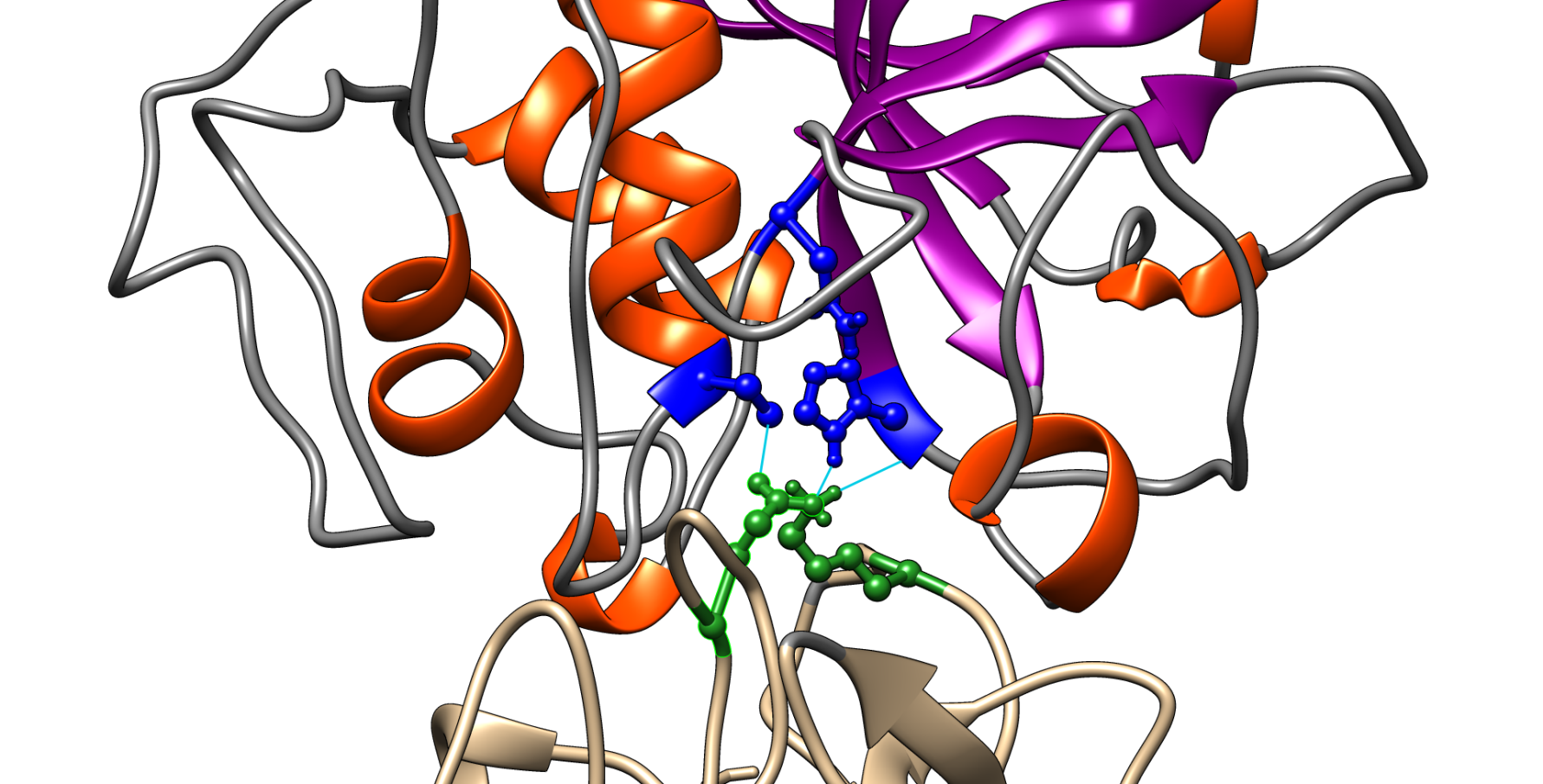


**(A)**

**Cys25**

**Asn175**

**His159**

**Cys25**

**Asn175**

**His159**

**Lys 164**

**Val165**

**Lys175**

**Glu122**

**Loop 9**

**Loop 9**

**Loop 6**


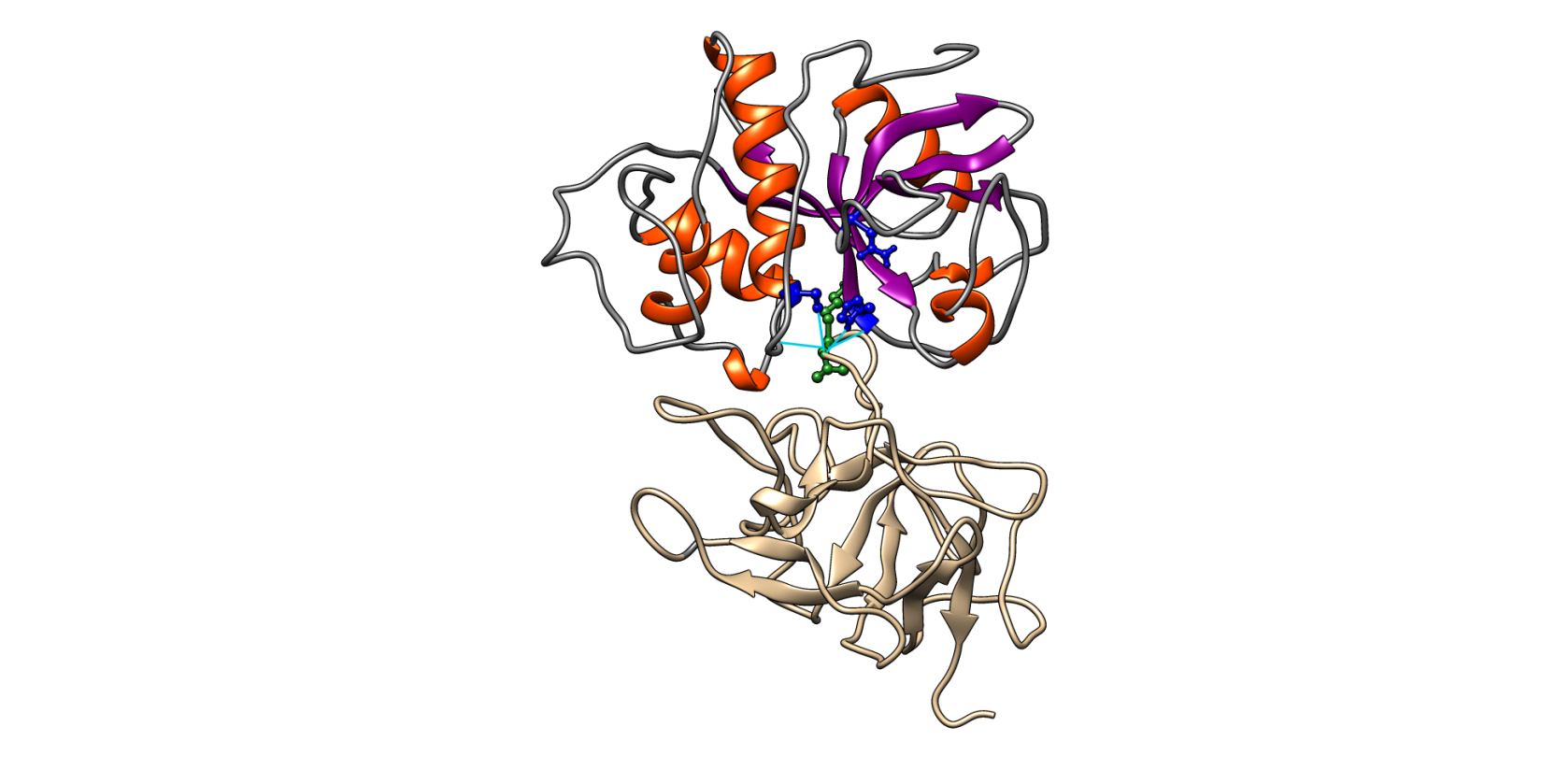


**(B)**

**Supplementary Figure 2.** Docking analysis of the interaction of papain with Arabidopsis Kunitz inhibitors. **(A)** AtKTI4. **(B)** AtKTI5. Secondary structures of papain are coloured in orange and violet, and its catalytic triad in blue. Arabidopsis Kunitz inhibitor is coloured in ochre and residues forming H-bonds with amino acids of the catalytic triad in green. Modelization and visualization were made by ClusPro server and Chimera tools.

| **Gene** | **Primer name** | **Sequence (5' 🡪 3')** | **Purpose** |
| --- | --- | --- | --- |
| *Ubiquitin* | UBQ-F | GCTCTTATCAAAGGACCTTCGG | RT-qPCR - Gene expression assays |
|  | UBQ-R | CGAACTTGAGGAGGTTGCAAAG |  |
| *AT1G73330* | KTI1-F | TGCATGAACGTTGGCATATT |  |
|  | KTI1-R | GAGTTGCTGCCTTCTGGAAC |  |
| *AT1G72290* | KTI2-F | TTCATCCAGCCGGTTAAGAC |  |
|  | KTI2-R | GGGAAGAAGTGTTTGGGTGA |  |
| *AT1G73325* | KTI3-F | CGAGTTTGATTCCACGGTTT |  |
|  | KTI3-R | ACCTTTCTGACCACCAATGC |  |
| *AT1G73260* | KTI4-F | TTCCCGAATCACAGAACCTC |  |
|  | KTI4-R | GCTTCCTCTCGTGGTCAAAC |  |
| *AT1G17860* | KTI5-F | ACTGGTTCAAGATCGACAAA |  |
|  | KTI5-R | TTTCCATCTTGCACAAACAC |  |
| *AT3G04320* | KTI6-F | ACAGCACATTCGCCATCCACA |  |
|  | KTI6-R | TATCGGATAGTCGTTGGTCAT |  |
| *AT3G04330* | KTI7-F | CCAGTACGTTCGCCATCGAGC |  |
|  | KTI7-R | CCTATATATTGGATAATCGTTGGATAA |  |
| *AY179605* | ACT-F | GATGGACAAGTCATCACCATTG |  |
|  | ACT-R | CTGAGGACAATGTTTCCGTACA |  |
| *AT1G73260* | KTI4-F | CGAATTCTGATGACAAAAACTACC | PCR - Cloning |
|  | KTI4-R | CTCGAGCTAAACATAGTCTTGGAC |  |
| *AT1G17860* | KTI5-F | GAATTCTGATGTCATCACTTCTC |  |
|  | KTI5-R | CTCGAGCTAAAGTATGCTCTTTTG |  |
| T-DNA | kti4.1-LP | TCGTTGCTATATGGTTGACGG | PCR - T-DNA insertion validation |
|  | kti4.1-RP | GCCAATGGTTATTTGTGGATG |  |
|  | kti4.2-LP | CATCCACAAATAACCATTGGC |  |
|  | kti4.2-RP | TCTACGCAGAATTTTTGCAAAC |  |
|  | kti5.1-LP | ATGACGTCGTCAAGCTTTGAC |  |
|  | kti5.1-RP | CCTGTAGGGGACGATAGGTTC |  |
|  | kti5.2-LP | TTCAACTTAGGGAATGCATGC |  |
|  | kti5.2-RP | TGATTTTGGTTAAATGCTCGG |  |
|  | LB | GCAATCAGCTGTTGCCCGTCTCACTGGTG |  |

**Supplementary Table 1.** Sequences of oligonucleotide used for qRT-PCR, PCR-cloning and validation of T-DNA insertion in the Salk lines.
